# Cell type-specific expression, regulation and compensation of CDKL5 activity in mouse brain

**DOI:** 10.1038/s41380-024-02434-7

**Published:** 2024-02-08

**Authors:** Margaux Silvestre, Kelvin Dempster, Simeon R. Mihaylov, Suzanne Claxton, Sila K. Ultanir

**Affiliations:** Kinases and Brain Development Laboratory, The Francis Crick Institute, London, UK

**Keywords:** Neuroscience, Autism spectrum disorders

## Abstract

CDKL5 is a brain-enriched serine/threonine kinase, associated with a profound developmental and epileptic encephalopathy called CDKL5 deficiency disorder (CDD). To design targeted therapies for CDD, it is essential to determine where CDKL5 is expressed and is active in the brain and test if compensatory mechanisms exist at cellular level. We generated conditional *Cdkl5* knockout mice in excitatory neurons, inhibitory neurons and astrocytes. To assess CDKL5 activity, we utilized a phosphospecific antibody for phosphorylated EB2, a well-known substrate of CDKL5. We found that CDKL5 and EB2 pS222 were prominent in excitatory and inhibitory neurons but were not detected in astrocytes. We observed that approximately 15–20% of EB2 pS222 remained in *Cdkl5* knockout brains and primary neurons. Surprisingly, the remaining phosphorylation was modulated by NMDA and PP1/PP2A in neuronal CDKL5 knockout cultures, indicating the presence of a compensating kinase. Using a screen of candidate kinases with highest homology to the CDKL5 kinase domain, we found that CDKL2 and ICK can phosphorylate EB2 S222 in HEK293T cells and in primary neurons. We then generated *Cdkl5/Cdkl2* dual knockout mice to directly test if CDKL2 phosphorylates EB2 in vivo and found that CDKL2 phosphorylates CDKL5 substrates in the brain. This study is the first indication that CDKL2 could potentially replace CDKL5 functions in the brain, alluding to novel therapeutic possibilities.

## Introduction

Cyclin-dependent kinase-like 5 (CDKL5) is a serine/threonine kinase and is a member of the CDKL family within the CMGC kinase group [1]. Mutations in the X-linked *CDKL5* cause a rare neurodevelopment disease called CDKL5 deficiency disorder (CDD, OMIM 300672, 300203 [2, 3]). CDKL5 mutations were initially classified as a variant of the Rett syndrome, but CDD is now recognized as a separate disorder [4–7]. It is characterized by early-onset intractable epilepsy, hypotonia, vision and speech impairments and profound neurodevelopment delays [2, 3]. Current treatment options for these patients are limited to supportive measures designed to mitigate the symptoms they experience. There are no therapies available that can rectify mutations in CDKL5 or effectively substitute for its absence. More than 265 pathogenic mutations were reported throughout *CDKL5* gene, including insertions/deletions, causing frameshift, and nonsense mutations [8]. Numerous missense mutations in the kinase domain of CDKL5 have been found to cause CDD [3, 8–10]. Several of these missense mutations result in reduced CDKL5 activity when expressed in cells [11], highlighting the importance of the kinase activity of CDKL5.

Ever since its initial identification, CDKL5 has been the subject of investigation in numerous studies, elucidating its intricate molecular functions [5, 12, 13]. In particular, efforts have been made to find substrates of the kinase that could highlight signaling pathways [11, 14, 15]. Using mass spectrometry, CDKL5 was found to phosphorylate microtubule binding proteins including microtubule-associated protein RP/end-binding Family Member 2 (MAPRE2 or EB2) on S222 [14]. EB2 was validated as a physiological substate using a phosphospecific antibody against EB2 S222 in *Cdkl5* knockout (KO) mice and CDD patient-derived neurons [14]. EB2 S222 phosphorylation (pS222) was subsequently successfully used to report CDKL5 activity in mouse and human models [14, 16, 17].

*Cdkl5* KO mouse models have been instrumental in advancing our understanding of CDKL5 functions [18–20]. Investigations employing cell-type specific conditional knockouts [21–25] have further enriched our insights, providing comprehensive characterizations spanning cellular mechanisms and behavioral outcomes. Collectively, CDKL5 deletions mirror many features observed in CDD. The animal models exhibit impairments in motor function, social interaction, learning and memory and in synaptic transmission [18, 19, 26–41]. Interestingly, re-expression of CDKL5 in adult *Cdkl5* KO mice was able to rescue the behavioral phenotypes [16]. In addition, a proof-of-concept study showed improvements in synaptic and behavioral deficits in *Cdkl5* KO mice by infecting the whole brain with AAV containing isoforms of *CDKL5* [42].

To develop gene replacement therapies for CDD, understanding CDKL5 activity patterns in the brain will be critical. CDKL5 is expressed in various tissues but it is highly enriched in forebrain structures such as the cortex, hippocampus, striatum, and olfactory bulb [18, 43, 44]. However, at the cellular level, findings are still inconsistent due to the lack of reliable CDKL5 antibodies for immunostaining. For example, Rusconi et al. showed that CDKL5 is expressed in glutamatergic and GABAergic neurons but not in glial cells [44, 45], whereas two other groups also found CDKL5 in glial cells [42, 46]. Moreover, finding other kinases that compensate for the loss of CDKL5 activity could also help with the development of new therapies. CDKL, RCK and GSK3 kinases share similar kinase domains with CDKL5 [1, 47], however it is unclear if they can phosphorylate similar substrates and compensate for each other.

In this study, we used EB2 pS222 as a functional readout to investigate the cell-type specificity of CDKL5 expression and the regulation of CDKL5 kinase activity. We also studied the compensation mechanisms of CDKL5-dependent phosphorylation in mouse brains. We found that CDKL5 is predominantly expressed and active in glutamatergic and GABAergic neurons but not in glial cells. We found that calcium influx via synaptic NMDA receptors, as well as PP1 and PP2A phosphatases, reduced EB2 phosphorylation. Furthermore, we noticed residual EB2 pS222 in absence of CDKL5, which was also regulated by the same signals. To determine whether another kinase could also phosphorylate CDKL5 substrates, we used a screen of candidate kinases that share high homology with CDKL5. We found that ICK and CDKL2 phosphorylate EB2 S222 in HEK293T cells and primary neurons. Due to the relatively low expression of ICK in the human and mouse brains, we focused on CDKL2. Our next objective was to validate the phosphorylation of CDKL5 substrates by CDKL2 under more physiological conditions. To achieve this, we established a novel mouse model with dual knockout of *Cdkl5* and *Cdkl2*. Through this model, we demonstrated in vivo phosphorylation of CDKL5 target proteins, EB2 and MAP1S, by CDKL2.

Our findings shed light on the cell type specificity of CDKL5 activity and improve our understanding of how CDKL5 activity is regulated and compensated for by other kinases. Importantly, we provide the first indication that CDKL2 could replace CDKL5 functions in the brain, which could lead to new therapeutic approaches.

## Materials and methods

### Animals

The mice were bred and handled according to the regulation of the animal (Scientific Procedures) Act 1986 of the United Kingdom, and approved by the Francis Crick Institute ethical committee. The mice were housed and maintained on a 12 h light/dark cycle and provided with food and water *ad libidum*. Each mouse strain was backcrossed into C57BL/6J genetic background.

#### Cdkl5 full body knockout mice

These mice were a generous gift from Cornelius Gross at the European Molecular Biology Laboratory in Rome [19]. Heterozygous females and wild-type males were used to maintain *Cdkl5* full KO mouse line and only the brains of male offspring were harvested to ensure *Cdkl5* wild type (WT) and hemizygous KO littermates.

#### Cdkl5 conditional knockout mice

In order to delete CDKL5 in specific cell types and generate *Cdkl5* conditional knockout mouse models, homozygous *Cdkl5* floxed females were crossed with heterozygous males Nex-Cre for excitatory neurons, Vgat-Cre for inhibitory neurons and Gfap-Cre for astrocytes. Brains were harvested at P20 from *Cdkl5* hemizygous floxed males expressing Cre and Cre-negative littermates as controls. *Cdkl5* floxed females were also a gift from Cornelius Gross [19]. Nex-Cre mouse model (Neurod6tm1(cre)Kan, MGI: 2668659 [48]) was a generous gift from Dr. Klaus Nave. Gfap-Cre mouse model (B6.Cg-Tg(Gfap-cre)77.6Mv; Stock Number: 012887, [49]) was purchased from Jackson Laboratories. Vgat-Cre (Slc32atm2(cre)Lowl/J, Stock Number: 016962 [50]) was a generous gift from Dr. Andreas Schaefer at the Francis Crick Institute.

#### Cre Ai14 mice

In order to verify the expression pattern of the Cre lines, Nex-cre, Dlx5,6-Cre and Gfap-Cre were crossed with Ai14 mice and litter mates were collected at P20. Ai14 mouse model (Gt(ROSA)26Sortm14(CAG-tdtomato)Hze; Stock Number: 007908) was purchased from Jackson Laboratories.

#### *Cdkl5/Cdkl2* knockout mice

This mouse model was generated by the Genetic Modification Service at the Francis Crick Institute using a strategy from Takea et al. [51]. A *Cdkl2* knockout strategy based on the deletion of exon 4 (ENSMUSE00000597281) was adopted, as ablation of this exon would lead to a frameshift and early translation stop of the *Cdkl2* transcript. CRISPR-Cas9 target sites were designed against regions of the upstream and downstream intronic sequence, using the WGE algorithm [52] to locate putative target sites within minimal off-target sites. Two sgRNAs with the following sequences were designed against intron 3: 5’- GCA CGC TCT CAA GCT ATG TG -3’ and 5’- TGC ACG CTC TCA AGC TAT GT-3’. Two other sgRNAs with the following sequences were designed against intron 4: 5’- GAA CTG CTG GTC GGT GAC GT -3’ and 5’- AAG TAT GGC AAG TAA GCG GC -3’. *Cdkl5* knockout (*Cdkl5*^tm1.2Cogr^) [19] embryos for modification were produced by in vitro fertilization (IVF) using heterozygous *Cdkl5* knockout females and hemizygous *Cdkl5* knockout males. Nine- to ten-week-old females were hyperovulated by intraperitoneal administration of 5IU/ female of CARD HyperOva® (2B Scientific, Cat # KYD-010-EX), and 46–48 h later, 5 IU/female of human chorionic gonadotropin (Chorulon®, MSD). Approximately 15–16 h later the females and males were sacrificed for oocyte and sperm collection and IVF; all performed according to the CARD protocol [51]. Seven hours post IVF, fertilized zygotes were electroporated in opti-MEM media containing 100 ng/µl total of the 4 sgRNAs and 600 ng/µl Cas9 (PURedit™ Cas9, Merck) using a Nepa21 electroporator (Nepagene) and a 50 µl electroporation slide (Nepa, CUY505P5). Four poring pulses were performed at 225 V, with a pulse width of 1 ms and pulse interval of 50 ms at positive polarity. These were followed by 5 transfer pulses at 20 V, with a pulse length of 50 ms and pulse interval of 50 ms with polarity switching. Electroporated zygotes were cultured overnight to two-cell stage and surgically reimplanted into recipient pseudopregnant CD1 females. Offspring were validated using PCR amplicons spanning the CRISPR/Cas9 target sites, followed by Sanger sequencing and zygosity was confirmed by qPCR. The genotype of *Cdkl2* KO and WT mice were determined from tissue biopsies using real time PCR with specific probes designed for each gene (Transnetyx, Cordova, TN).

### Cell cultures

#### HEK293T cells

The cells were grown in Dulbecco’s modified Eagle medium (DMEM) supplemented with FBS and penicillin/streptomycin at 37 °C with 5% CO_2_ for maximum 25 passages. Each passage consisted in dissociating the cells using 0.25% trypsin-EDTA for 3 min at 37 °C and adding 1:10 of the cells into a new T75 flask. This step was repeated every 3-4 days.

#### Cdkl5 WT and KO primary cultures

Brains from individual male E16.5 embryos from heterozygous *Cdkl5* KO mothers were collected and placed in hibernate buffer (Gibco) while the *Cdkl5* WT and KO genotype was determined. An equal number of both genotypes was selected, and cortices and hippocampi from those embryos were then dissected and individually washed three times with Hank’s balanced salt solution (HBSS). An incubation with 0.25% trypsin for 15 min at 37 °C was followed by four times washing with HBSS. Cells were dissociated and then counted using a hemocytometer. Neurons were plated on 18 mm glass coverslips (Fisher) or directly on the plastic well of 12-well culture plates at a density between 150,000 and 300,000 cells. The coverslips and the wells were coated with 0.1 M borate buffer containing 60 µg/mL poly-D-lysine and 2.5 µg/mL laminin, and placed in the incubator overnight. Neurons were plated with minimum essential medium (MEM) containing 10% fetal bovine serum (FBS), 0.5% dextrose, 0.11 mg/mL sodium pyruvate, 2 mM glutamine and penicillin/streptomycin. After 4 h, cultures were transferred to neurobasal medium containing 1 mL of B27 (Gibco), 0.5 mM GlutaMAX, 0.5 mM glutamine, 12.5 µM glutamate and penicillin/streptomycin. Primary neuronal cultures were kept at 37 °C and 5% CO_2_. Every 3-4 days, 20-30% of the maintenance media was refreshed.

#### Rat primary cultures

Pregnant Long Evans rats were ordered from Jackson. E18.5 rat embryos were removed from the uterus and the brains were taken out. Cortices were dissected out, pooled from multiple animals and washed three times with HBSS. The rest of the protocol is identical to the one used for the mouse primary neurons.

### DNA constructs

pTriex6-StrepII-EB2 vector were already described in Baltussen, Negraes et al. [14]. hCDKL1, hCDKL2, hCDKL3, hMAK and hMOK were purchased in a pDONR223 vector from Addgene (respectively #23389, #23421, #23628, 23624 and #23645) and were transferred into a pCAG-DEST-3xFLAG-TwinStrep vector using the Gateway cloning method (Invitrogen). hCDKL4 (NM_001346911.1) and hICK (NM_014920.4) were purchased in a pcDNA3.1 + /C-(K)-DYK vector from Genscript and were transferred into a pCAG-DEST-3xFLAG-TwinStrep vector using the Gateway cloning method (Invitrogen). hCDKL5 (NM_001323289.1) in a pcDNA3-FLAG vector was generously donated by Charlotte Kilstrup-Nielsen at the University of Insubria and was then transferred in a pCAG-DEST-3xFLAG-TwinStrep vector using the Gateway cloning method (Invitrogen). The final plasmids were respectively called CDKL1-FLAG WT, CDKL2-FLAG WT, CDKL3-FLAG WT, MAK-FLAG WT, MOK-FLAG WT, CDKL4-FLAG WT, ICK-FLAG WT and CDKL5-FLAG WT. In order to generate kinase dead plasmids, K33 34 36R for ICK, K33R for CDKL2 and K42R D153A for CDKL5 mutations were generated using site-directed mutagenesis. The final plasmids were respectively called ICK-FLAG KD, CDKL2-FLAG KD and CDKL5-FLAG KD.

### Western blotting

Mouse brain tissue or neuronal culture were lysed in 1X sample buffer (Invitrogen) containing 0.1 M DTT. Lysates were sonicated briefly eight times, for the cortices, or twice, for the cultures, and denatured at 70 °C for 10 min. The samples were centrifuged at 13,300 rpm for 10 min and ran on NuPage 4–12% Bis-Tris polyacrylamide gels (Invitrogen). Proteins were transferred onto a Immobilon PVDF membrane (Millipore), which was then blocked in 4% milk in tris-buffered saline containing 0.1% Tween-20 (TBST) for 30 min. Primary antibodies were incubated at 4 °C overnight, and HRP-conjugated secondary antibodies at room temperature (RT) for 2 h. The following primary antibodies were used: rabbit anti-CDKL5 (1:1,000, Atlas HPA002847), rabbit anti-EB2 pS222 (1:2,000; Covalab; previously described [14]), rabbit anti-MAP1S S812 (1:500; Covalab; previously described [14]), rat anti-EB2 (1:2,000; Abcam ab45767), rabbit anti-MAP1S (1:500; Atlas Antibodies HPA050934), mouse anti-tubulin (1:100,000; Sigma T9026), rabbit anti-CDKL2 (1:1,000; customed made for this study by Covalab; peptide sequence: “KIKDSKVFKVKGSKIDVE” [mouse] and “QKDARNVSLSKKSQNRKK” [human]). The following secondary antibodies were used at a concentration of 1:10,000: HRP-conjugated anti-rabbit (Jackson 711-035-152), HRP-conjugated anti-mouse (Jackson 715-035-151) and HRP-conjugated anti-rat (Jackson 712-035-153). The membrane was developed using ECL reagent (Cytiva) and was visualized with an Amersham Imager 600 or ImageQuant 800 (GE Healthcare). Quantification of Western blots was manually performed using Image Studio Lite Software (version 5.2). EB2 phosphorylation was measured relative to total EB2 and the other proteins were normalized to tubulin, if not indicated otherwise.

### Immunocytochemistry

#### In mouse primary neurons

DIV8 cultured mouse neurons on glass coverslips were fixed with 1 mM EGTA-Methanol for 5 min at −20 °C, followed by 10 min with 4% PFA-4% sucrose in PBS. Neurons were blocked for 1 hour at RT with 10% normal goat serum (NGS) in 0.1% Triton-PBS. They were then incubated with primary antibodies overnight at 4 °C, washed three times with PBS and incubated with secondary antibodies at RT for 2 h. Coverslips were again washed three times, of which the second contained 1:2,000 DAPI stain (Thermo Scientific). Coverslips were mounted with Fluoromount-G (Southern Biotech) on glass slides. The following primary antibodies were used: mouse anti-GAD67 (1:2,000 – Millipore MAB5406), chicken anti-GFAP (1:1,000 – Abcam ab134436), mouse anti-FLAG (1:2,000 – Sigma F1804), rabbit anti-EB2 pS222 (1:2,000; Covalab; previously described [14]) and rat anti-EB2 (1:1,000 – Abcam ab45767). The following secondary antibodies were used at a concentration of 1:500: Alex Fluor 568 anti-rabbit (Life technologies A10042), Cy5-conjugated anti-rat (Jackson 712-175-153), Alex Fluor 488 anti-chicken (Thermo Fisher A32931) and Alex Fluor 488 anti-mouse (Jackson 715-545-151). Images were acquired with Leica MPSP5 or MPSP8 Upright Confocal (63x/1.4 oil or 40x/1.25-0.75, 0.2 μm zstep size). For the cell-type specificity experiment, the mean intensities of total EB2 and EB2 pS222 in GAD67 or GFAP positive cells were measured using a custom Fiji plugin. For the kinases experiment, the cell bodies of FLAG-positive or negative cells were drawn manually and the mean intensities of total EB2 and EB2 pS222 were measured in those areas. The final mean fluorescence was calculated as the ratio between mean fluorescence of EB2 pS222 and mean fluorescence of total EB2.

#### In Cre Ai14 brain sections

Sagittal sections were prepared from the PFA fixed brains with a Leica VT1000S vibrating blade microtome at 40 µm. Sections were washed three times in PBS, of which the second contained 1:2,000 DAPI stain (Thermo Scientific). Sections were mounted on slides with Fluoromount (Southern Biotech). Images were acquired with Olympus IX83 P22F (camera: Hamamatsu ORCA-Flash4.0; software: OLYMPUS cellSens Dimension 1.13 (Build 13479)).

### Transfection

#### HEK293T cells

Cells were split into 12-well plates and, 24 h after the last passage, cells were transfected with 3 µL of Xtremegene 9 (Roche) and 1 µg of DNA for 48 h at 37 °C. Cells were then washed once with cold PBS containing proteases and phosphatases inhibitors (1X protease inhibitor cocktail (Roche), 1:100 phosphatase inhibitor III (Roche), 1X Pierce phosphatase Inhibitor (Thermo Scientific), and 0.5 µM of okadaic acid (Fluorchem)) and were lysed directly in 300 µL of 1X sample buffer supplemented with 0.1 M DTT.

#### Mouse primary neurons

Cells were plated on glass coverslips in 12-well plates and each well was transfected on the 4^th^ day in vitro (DIV4) with 3 µL of Lipofectamine 2000 (Thermo Scientific) and 1 µg of DNA for 15 min at 37 °C. Before transfection, half of the maintenance media was taken off and was used to replace the media after transfection.

### Neuronal treatment

Cortical primary rat neurons or *Cdkl5* cortical primary neurons were plated onto 12-well plates and treated by adding directly the treatment to the media. The neurons were then incubated at 37 °C before being lysed in 300 µL of 1X sample buffer (Invitrogen) containing 0.1 M DTT. The final concentration and the length of the treatment are indicated in the result sections. The zero-time point is a control condition where water or DMSO was added to the well for the longest time point of the assay. For the NMDA treatment in absence of calcium, the medium of the neurons was changed to artificial cerebrospinal fluid (ACSF) buffer (125 mM NaCl, 2.5 mM KCl, 26 mM NaHCO_3_, 1.25 mM NaH_2_CO_3_, 25 mM glucose, 2 mM CaCl_2_ and 1 mM MgCl_2_) or to Ca^2+^-free ACSF buffer (125 mM NaCl, 2.5 mM KCl, 26 mM NaHCO_3_, 1.25 mM NaH_2_CO_3_, 25 mM glucose, 10 mM EGTA and 1 mM MgCl_2_). The neurons were then treated and lysed the same way. For the inhibition of synaptic NMDA receptors, the neurons were pre-incubated for 10 min with 10 µM MK-801 (Tocris) and 50 µM bicuculline (Tocris), then washed with fresh new media, treated with 50 µM NMDA and finally lysed.

### Single-cell RNA sequencing data

*Mouse* - The trimmed mean gene expression aggregated per cell type from the *whole cortex & hippocampus – 10X genomics (2020) with 10X-SMART-SEQ taxonomy* database was downloaded on the Allen Brain Map website [53, 54]. In this database, they were able to profile around 1.3 million cells covering multiple cortical areas and the hippocampus, and then derived a transcriptomic cell type taxonomy revealing glutamatergic neuronal, GABAergic neuronal and non-neuronal classes. Each class contains a certain number of clusters that regroup cells with a similar gene expression pattern. For the purpose of this project, the different clusters were regrouped by classes; glutamatergic neurons, GABAergic neurons and non-neuronal cells. The mRNA trimmed mean expression of *Cdkl5*, *Eb2, Nex, Gfap, Vgat, Gad67, Ick, Mak, Mok, Cdkl1, Cdkl2, Cdkl3* and *Cdkl4* were extracted for those clusters and their mRNA expressions were represented on a heatmap, with each rectangle representing a cluster using a custom script in MATLAB (version R2021b).

*Human* - The trimmed mean gene expression aggregated per cell type from the *Multiple cortical areas – SMART-Seq (2019)* database was downloaded on the Allen Brain Map website [55, 56]. In order to generate this database, the authors dissociated and sorted nuclei from postmortem and neurosurgical donor brains (middle temporal gyrus only) using the neuronal marker NeuN. Expression was then profiled using SMART-Seq v4 or 10x RNA-sequencing and nuclei were grouped intro clusters. For the purpose of this project, the different clusters were regrouped by classes; glutamatergic neurons, GABAergic neurons and non-neuronal cells. The mRNA trimmed mean expression of *ICK, MAK, MOK, CDKL1, CDKL2, CDKL3, CDKL4* and *CDKL5* were extracted for those clusters and their mRNA expressions were represented on a heatmap, with each rectangle representing a cluster using a custom script in MATLAB (version R2021b).

### Statistics

Data was analyzed using GraphPad Prism 9. Exact values of n and statistical methods are mentioned in the figure legends. A *p*-value higher than 0.05 was not considered as statistically significant. Thresholds for significance were placed at *p ≤ 0.05, **p ≤ 0.01, ***p ≤ 0.001 and ****p ≤ 0.0001. All errors bars in the figures are standard deviation (SD), except when mentioned otherwise. Errors bars and non-significant statistics were not indicated, except when mentioned otherwise.

## Results

### CDKL5 is expressed and active in excitatory and inhibitory neurons

CDKL5 is enriched in the brain during early postnatal development but its cell-type specificity is debated. Here, we took a multidisciplinary approach where we used *Cdkl5* conditional knockout (cKO) mouse models, *Cdkl5* KO neuronal culture and publicly available single-cell RNA sequencing database to determine the localization of CDKL5 expression and activity in the brain.

**Fig. 1:**
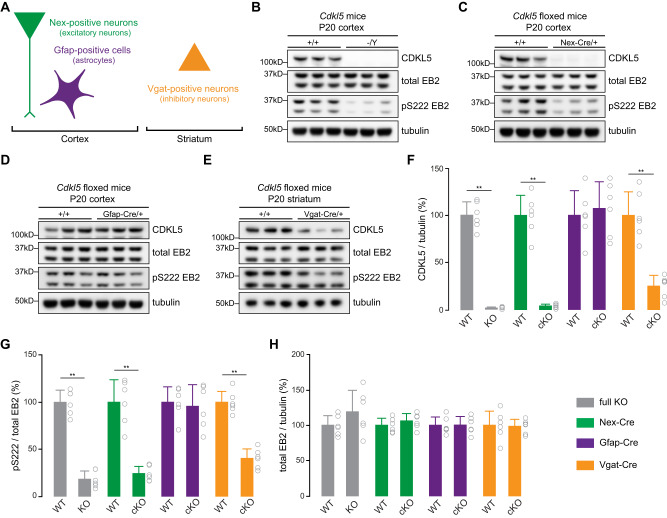
CDKL5 is expressed and active in excitatory and inhibitory neurons of 20-day old mice brains. **A** Using a Cre-Lox recombination system, CDKL5 was selectively knocked out in excitatory neurons (Nex-Cre; green), in inhibitory neurons (Vgat-Cre; orange), in astrocytes (Gfap-Cre; purple), or in every cell type (*Cdkl5* full KO; gray) and cortex or striatum were collected at P20. **B**–**E** Western blots showing expression of CDKL5, total EB2 and tubulin, and level of EB2 S222 phosphorylation (pS222) in, respectively, *Cdkl5* full KO, Nex-Cre cKO, Gfap-Cre cKO and Vgat-Cre cKO mice at P20. **F**–**H** Quantification of CDKL5 expression, EB2 pS222 and total EB2 expression in P20 *Cdkl5* full KO, Nex-Cre cKO, Gfap-Cre cKO and Vgat-Cre cKO mice. Mann–Whitney test. *n* = 3 animals per genotype with 2 technical replicates. **p ≤ 0.01. *p ≤ 0.05.

We started by crossing *Cdkl5* floxed females with males that express Cre recombinase under the control of *Nex (NeuroD6)* promoter for deletion in excitatory neurons, *Gfap* promoter for astrocytes as well as *Vgat* (*Slc32a1*) promoter for inhibitory neurons (Fig. 1A). Cre expressing mice were crossed with an Ai14 reporter mouse line, which confirmed that, for each promoter, the Cre recombination occurred in the expected regions of postnatal day 20 (P20) brains (Supplementary Fig. S1A–C), as shown in previous studies [48–50]. We collected the brain regions, where Cre-expressing cells were abundant: we used the cerebral cortex for Nex-Cre cKOs and Gfap-Cre cKOs and striatum from Vgat-Cre cKOs, as GABAergic neurons account for 95% of striatal neurons in rodents [57]. We also used *Cdkl5* full body knockout (full KO) mice as a comparison. Using Western blots, we analyzed CDKL5 protein and EB2 phosphorylation levels (Fig. 1B–E). In Nex-Cre cKO mice, CDKL5 and EB2 phosphorylation levels were significantly reduced indicating a prominent presence of CDKL5 activity in cortical pyramidal neurons (Fig. 1F, G). When CDKL5 was knocked out in astrocytes using Gfap-Cre cKO mice, no significant change was observed in CDKL5 expression and EB2 phosphorylation levels (Fig. 1F, G). The Vgat-Cre cKO animals showed a significant reduction of CDKL5 expression and EB2 phosphorylation, indicating the presence of CDKL5 activity in inhibitory neurons (Fig. 1F, G). As expected, in full *Cdkl5* KO mice, CDKL5 expression was absent (Fig. 1F) and EB2 pS222 was drastically reduced to 17.8% of control animals (Fig. 1G). Finally, no changes in total EB2 expression were found in full KO or cKO mouse models (Fig. 1H).

**Fig. 2:**
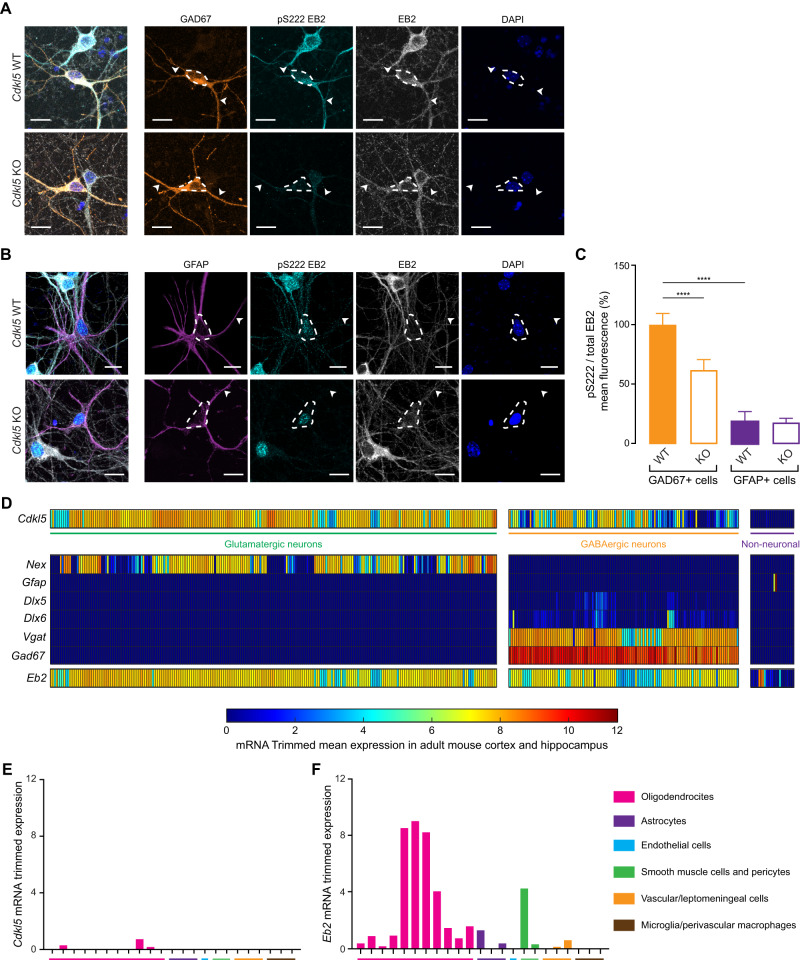
EB2 phosphorylation is present in inhibitory neurons but not in astrocytes of mouse primary neurons. **A** *Cdkl5* KO and WT cortical neurons at DIV8 in culture were co-stained with GAD67, an inhibitory neuron marker, EB2 pS222, total EB2 and DAPI. White arrows indicate neuronal dendrites and dashed lines indicates cell body. Scale bar is 15 μm. **B** *Cdkl5* KO and WT cortical neurons at DIV8 in culture were co-stained with GFAP, an astrocyte marker, EB2 pS222, total EB2 and DAPI. Scale bar is 15 μm. Dashed lines indicates cell body. **C** Quantification of EB2 phosphorylation mean fluorescence in GAD67- and GFAP-positive cells of *Cdkl5* WT and KO cortical neurons. *n* = 11–16 cells. Mann–Whitney test. ****p ≤ 0.0001. **D** Expression of *Cdkl5* and different cell markers in mouse adult isocortex and hippocampus from single-cell RNA sequencing data. **E**, **F** Closer view of the expression of *Cdkl5* and *Eb2* respectively in different non-neuronal cells of adult mouse isocortex and hippocampus from single-cell RNA sequencing data.

EB2 phosphorylation has been used as a reliable readout of CDKL5 activity [16, 17]. Moreover, EB2 phosphospecific antibodies can be used in immunostainings as their specificity has been validated in *Cdkl5* KO cultures [14]. To validate the observations outlined above, *Cdkl5* wild type (WT) and KO cortical primary cultures were fixed at Days in vitro 8 (DIV8) and stained with GAD67 (a GABAergic neuronal marker) or GFAP (an astrocyte marker) together with anti-phosphospecific EB2 S222, total EB2 and DAPI (Fig. 2A, B). The mean fluorescence of EB2 phosphorylation and total EB2 were measured and quantified in GAD67-positive or GFAP-positive cells (Fig. 2C). A significant reduction of 38% of EB2 pS222 was observed in GAD67-positive cells of *Cdkl5* KO primary culture compared to WT. However, no significant change was found in GFAP-positive cells, further supporting the absence of CDKL5 activity in astrocytes (Fig. 2C). To conclude, these results confirm the presence of EB2 phosphorylation in GABAergic neurons but not in GFAP-positive astrocytes in cultures.

We next analyzed mRNA expression in mouse cerebral cortex and hippocampus from a publicly available single-cell RNA sequencing database, where the transcriptomic data is classified in glutamatergic neuronal, GABAergic neuronal and non-neuronal classes [53, 54]. Each class contains a certain number of clusters that group cells with a similar gene expression pattern. For the purpose of this analysis, we regrouped the different clusters by classes; glutamatergic neurons, GABAergic neurons and non-neuronal cells and we then extracted mRNA trimmed mean expression of *Cdkl5* and different cell-type markers from those clusters. We represented the mRNA expressions on a heatmap, with each rectangle representing a cluster (Fig. 2D). *Cdkl5* was found to be highly expressed in glutamatergic and GABAergic neurons (Fig. 2D). Moreover, in non-neuronal cells, *Cdkl5* seemed to be expressed at a very low level in only three clusters of oligodendrocytes (Fig. 2D, E). We also investigated the mRNA level of *Eb2* and found that it was expressed in glutamatergic and GABAergic neurons (Fig. 2D). *Eb2* was also expressed in multiple non-neuronal clusters including oligodendrocytes and smooth muscle cells/pericytes at a relatively high level. However, *Eb2* expression is very low in astrocytes, vascular/leptomeningeal cells and microglia/perivascular macrophages clusters (Fig. 2D, F).

In summary, we used three distinct methods in order to dissect the cell-type of CDKL5 expression and activity. We found that CDKL5 is predominantly expressed in glutamatergic and GABAergic neurons but not in astrocytes. Moreover, EB2 phosphorylation closely follows CDKL5’s expression pattern, and is present in excitatory and inhibitory neurons but not in astrocytes.

### CDKL5 activity is downregulated by the influx of calcium through synaptic NMDA receptors

**Fig. 3:**
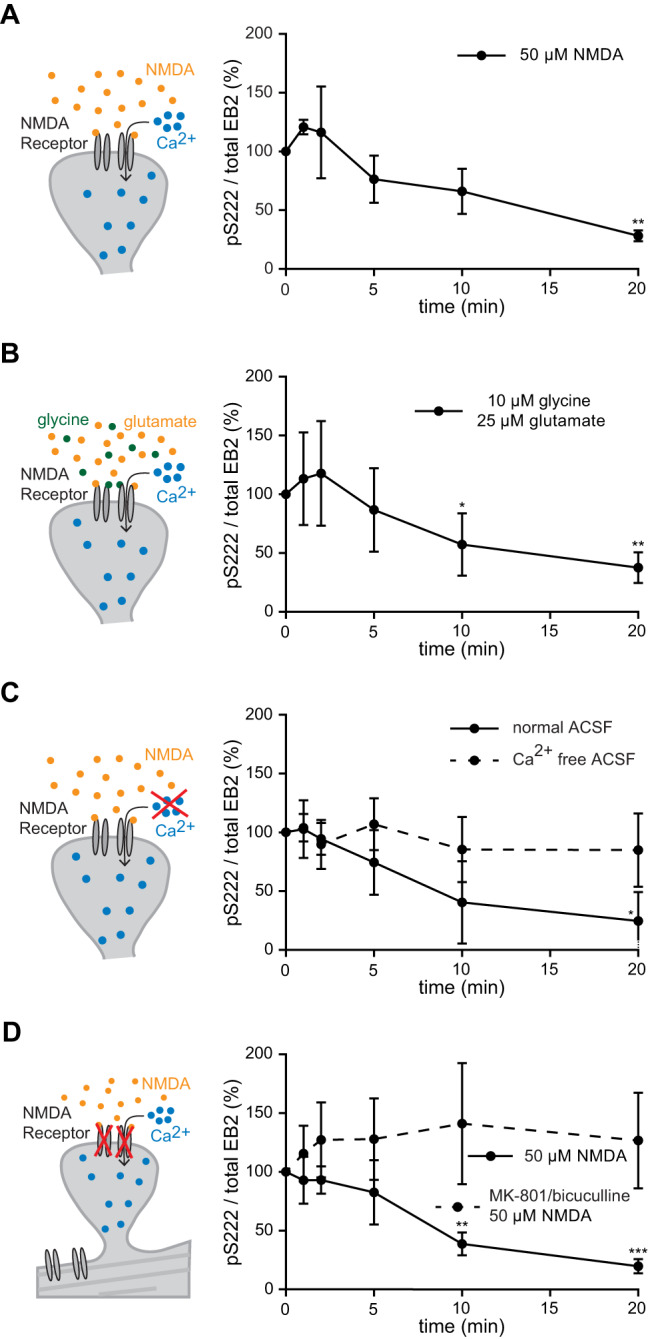
EB2 S222 phosphorylation is reduced by synaptic NMDAR-dependent calcium influx. **A** Quantification of EB2 pS222 levels upon 50 μM NMDA treatment of DIV14 rat primary cortical neurons. Welch’s *t* test. *n* = 3 technical replicates. **B** Quantification of EB2 pS222 levels upon 10 μM glycine and 25 μM glutamate treatment of DIV15 rat primary cortical neurons. Welch’s *t* test. *n* = 4 technical replicates. **C** Quantification of EB2 pS222 levels upon 50 μM NMDA treatment of DIV15 rat primary cortical neurons in normal ACSF and in calcium free ACSF. Dunnett’s multiple comparisons test. *n* = 4 technical replicates. **D** Quantification of EB2 pS222 levels upon 10 μM MK801 and 50 μM bicuculline pre-treatment (10 min) and 50 μM NMDA treatment of DIV15 rat primary cortical neurons. Dunnett’s multiple comparisons test. *n* = 4 technical replicates.

Despite early evidence indicating CDKL5’s regulation by neuronal activity [14, 58], it is not known how CDKL5 substrate phosphorylation is regulated. Having established that CDKL5 activity predominantly localizing within neurons, we next investigated the regulatory mechanisms upstream of CDKL5 within primary neuronal cultures. We treated DIV14 rat primary neurons with 50 µM of NMDA for up to 20 min and examined the level of EB2 phosphorylation using Western blotting (Fig S2A). A significant reduction of EB2 pS222 level was observed after 20 min of treatment (Fig. 3A). The same reduction was found when neurons were treated with 10 µM of glycine and 25 µM of glutamate, another treatment that induces NMDA receptor (NMDAR) activation [59, 60] (Fig. 3B and Supplementary Fig. S2D). These findings suggest that NMDA-dependent neuronal activity downregulates CDKL5 activity. We next tested whether the reduction of EB2 phosphorylation level upon NMDAR activation is dependent on calcium entry. For this, media of DIV15 primary rat neurons were changed to an extracellular medium depleted of calcium, and neurons were then treated with NMDA for up to 20 min. No significant reduction of EB2 pS222 was observed in the absence of calcium (Fig. 3C and Supplementary Fig. S2G).

We next tested if the modulation of CDKL5 activity through NMDARs is induced by synaptic or extrasynaptic receptors [61]. We used a pharmacological strategy, where a blocker of active NMDARs (MK-801) was added to the neurons while synaptic NMDA receptors were stimulated by reducing synaptic inhibition via blocking GABAergic receptors with bicuculine in primary cultures. After 10 min, when synaptic NMDARs were inactivated, neurons were treated with NMDA and the level of EB2 phosphorylation was examined by Western blotting (Supplementary Fig S2J). Upon pharmacological block of synaptic NMDA receptors, no significant change in EB2 pS222 was observed (Supplementary Fig. 3D), indicating that synaptic NMDARs are required for the calcium-dependent reduction of EB2 phosphorylation. We also assessed the levels of total EB2 and CDKL5 in order to confirm that the changes in phosphorylation are not due to the changes of their expression. No significant changes were found in total EB2 or CDKL5 levels after the different neuronal treatments (Supplementary Fig. S2B–C, E, F, H, I and K–L).

Our data shows that CDKL5 activity is downregulated by neuronal activity in an NMDA-dependent manner, and it reveals that synaptic NMDA receptors activation and entry of calcium are necessary to induce this modulation of CDKL5 activity.

### EB2 S222 is dephosphorylated by PP1 and PP2A phosphatases

**Fig. 4:**
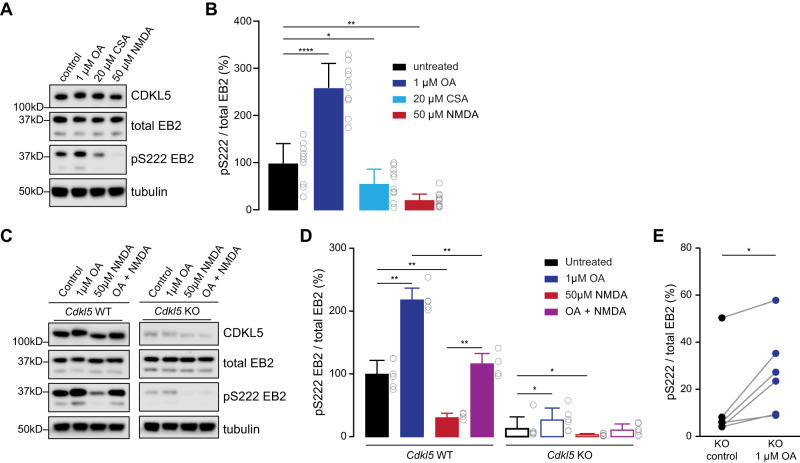
Inhibition of PP1 and PP2A increases EB2 phosphorylation in presence and absence of CDKL5. **A** Western blots showing CDKL5, total EB2 and tubulin expression and EB2 pS222 level upon 1 μM okadaic acid (OA) for 40 min, 20 μM cyclosporin A (CSA) for 40 min and 50 μM NMDA treatment for 20 min in DIV14-15 rat primary cortical culture. **B** Quantification of EB2 pS222 level upon 1 μM OA for 40 min, 20 μM CSA for 40 min and 50 μM NMDA treatment for 20 min in DIV14-15 rat cortical primary culture. Mann–Whitney test. *n* = 5 with 2 technical replicates per treatment. ****p < 0.0001. **p ≤ 0.01. *p ≤ 0.05. **C** Western blots showing total EB2 and tubulin expression and EB2 pS222 level upon 1 μM OA treatment for 40 min, 50 μM NMDA treatment for 20 min and a combination of OA and NMDA treatments (1 μM OA treatment for 20 min followed by 20 min of OA + NMDA) in DIV8 mouse *Cdkl5* WT and KO primary cortical culture. **D** Quantification of EB2 S222 phosphorylation level upon 1 μM OA treatment for 40 min, 50 μM NMDA treatment for 20 min and a combination of OA and NMDA treatments in DIV8 mouse *Cdkl5* WT and KO primary cortical culture. Mann–Whitney test. *n* = 3 with 2 technical replicates per treatment. **p ≤ 0.01. *p ≤ 0.05. Only significance within WT treatments and within KO treatments are shown. **E** Pairwise quantification of EB2 S222 phosphorylation upon 1 μM OA treatment for 40 min in *Cdkl5* KO primary cortical culture. Mann–Whitney test. *n* = 3 with 2 technical replicates per treatment. *p ≤ 0.05.

Protein phosphorylation exist in a balance that involves kinases as well as phosphatases which remove phosphate groups from the target protein. Very little is known about the mechanism of EB2 dephosphorylation. To address this gap, we treated rat primary neurons with specific phosphatase inhibitors, and assessed the levels of EB2 phosphorylation by Western blotting, expecting an increase of EB2 pS222 if the residue is dephosphorylated by that phosphatase. Using okadaic acid (OA) as an inhibitor of PP1 and PP2A phosphatases and cyclosporin A (CSA) as an inhibitor of calcineurin phosphatases, we found that the level of EB2 pS222 was increased 2.7 folds upon 1 µM of OA treatment for 40 min (Fig. 4A, B). In contrast, 20 µM of CSA treatment for 40 min showed a small but significant decrease of EB2 pS222 (Fig. 4A, B), which may be due to an effect of CSA on CDKL5’s activity. As a control, neurons were treated with NMDA and as expected, the level of EB2 pS222 was reduced (Fig. 4B). We also assessed the expression of total EB2 and CDKL5 and no changes were observed except for a significant reduction in total EB2 in neurons treated with OA when compared to the control condition (Supplementary Fig. S3A, B).

Overall, our experiments showed that EB2 S222 is dephosphorylated by PP1 and PP2A phosphatases but not by calcineurin. This result suggests that CDKL5 and PP1/PP2A maintain the basal level of EB2 phosphorylation in neurons.

### The increase of EB2 phosphorylation upon PP1 and PP2A inhibition is still present in the absence of CDKL5

We had observed that EB2 phosphorylation did not completely disappear in *Cdkl5* full KO animals as approximately 18% of the EB2 pS222 signal was remaining (Fig. 1B, G). Although we could not rule out that the remaining signal was due to a background of the phosphospecific antibody, we hypothesized that EB2 could potentially be phosphorylated by other kinases in addition to CDKL5. We decided to test this by taking advantage of the OA-dependent regulation of EB2 pS222 and asked if EB2 phosphorylation could be regulated in absence of CDKL5. We prepared *Cdkl5* WT and KO mouse neurons from littermate embryos and treated them with OA or NMDA at DIV8. WT neurons confirmed the results obtained earlier in rat neurons, showing EB2 phosphorylation was significantly increased upon OA treatment and reduced upon NMDA (Fig. 4C, D). Interestingly, in KO neurons, we also observed a significant increase in EB2 pS222 upon OA treatment and a reduction upon NMDA treatment (Fig. 4C–E). This finding implies that, even in the absence of CDKL5, another NMDA-regulated kinase is capable of phosphorylating EB2. Furthermore, when cultures were treated with OA for 20 min followed by OA + NMDA for 20 min (OA + NMDA), EB2 phosphorylation levels resembled those of control conditions (Fig. 4C, D). This observation reinforces the notion that NMDA continues to regulate EB2 phosphorylation even when phosphatases are inactive, affirming the role of another NMDA-regulated kinase. CDKL5 was largely absent in KO cultures, as expected (Supplementary Fig. S4A), and a significant increase of EB2 expression was observed in *Cdkl5* KO primary mouse neurons (Supplementary Fig. S4C), indicating a potential compensatory mechanism. There were no changes in total EB2 or CDKL5 expression across the different treatments compared to control condition (Supplementary Fig. S4B, D).

To summarize, we observed an increase of EB2 phosphorylation upon OA treatment and a decrease of EB2 phosphorylation upon NMDA treatment in absence of CDKL5. This result indicates that one or several other kinases, that are also regulated by NMDA receptor activation, might phosphorylate EB2.

### ICK and CDKL2 kinases can phosphorylate EB2 in HEK293T cells and in primary neurons

**Fig. 5:**
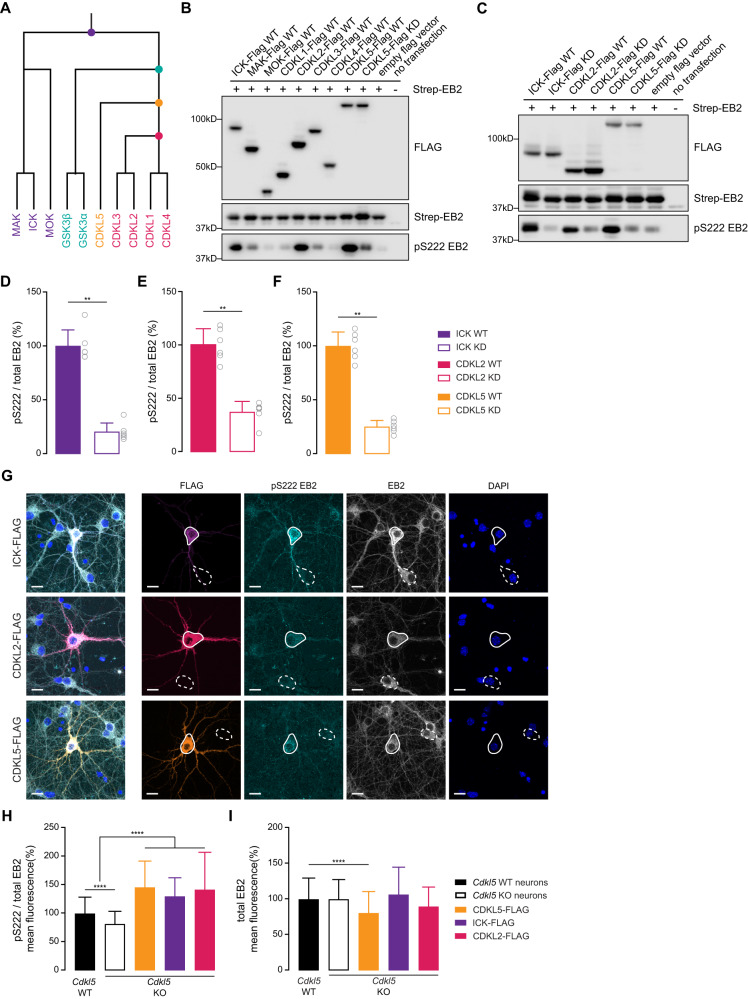
EB2 phosphorylation by ICK and CDKL2 in HEK293T cells and primary neurons. **A** CDKL5 and its closely-related kinases on the human phylogenetic tree. **B** Western blot showing levels of FLAG, total EB2 and EB2 pS222 in HEK293T cells co-transfected with StrepII-tagged EB2 and FLAG-tagged ICK WT, MAK WT, MOK WT, CDKL1 WT, CDKL2 WT, CDKL3 WT, CDKL4 WT, CDKL5 WT or CDKL5 kinases-dead (KD). **C** Western blot showing levels of FLAG, total EB2 and EB2 pS222 in HEK293T cells co-transfected with StrepII-tagged EB2 and FLAG-tagged ICK WT/KD, CDKL2 WT/KD or CDKL5 WT/KD. **D**–**F** EB2 pS222 quantification comparing WT and KD of ICK, CDKL2 and CDKL5 expressing cells respectively. *n* = 6 per condition. Mann–Whitney test. **p ≤ 0.01. **G** DIV8 *Cdkl5* KO primary mouse hippocampal neurons, overexpressing FLAG-tagged ICK, CDKL2 or CDKL5 WT, were co-stained with FLAG, EB2 pS222, total EB2 and DAPI. Full lines highlight neurons expressing the kinases and dashed lines highlight neurons which do not express the kinases. Scale bar is 30 μm. **H** Quantification of EB2 pS222 mean fluorescence in *Cdkl5* WT neurons, in *Cdkl5* KO neurons and in *Cdkl5* KO neurons overexpressing the three kinases. *n* = 21–166 per condition. Mann–Whitney test. ****p ≤ 0.0001. **I** Quantification of total EB2 mean fluorescence in *Cdkl5* WT neurons, in *Cdkl5* KO neurons and in *Cdkl5* KO neurons overexpressing the three kinases. *n* = 21–166 per condition. Mann–Whitney test. ****p ≤ 0.0001.

In order to determine which kinase(s) can phosphorylate EB2 S222, we performed a small screen using kinases closely related to CDKL5 in HEK293T cells. CDKL5 kinase domain has high similarity with RCK kinases, GSK3 kinases and CDKL kinases (Fig. 5A). GSK3s are well-studied kinases, and their consensus sequence is distinct from CDKL5 consensus motif (RPXS*) [62], therefore we decided to focus on RCK kinases and CDKL kinases. We co-expressed FLAG-tagged kinases with StrepII-tagged EB2 in HEK293T cells and assessed the level of EB2 phosphorylation by Western blotting (Fig. 5B). As expected, EB2 was strongly phosphorylated by CDKL5 WT and not by CDKL5 kinase dead mutant (KD) (Fig. 5B). The remaining signal in KD might be due to endogenous kinases phosphorylating EB2 S222. Interestingly, HEK293T cells expressing ICK and CDKL2 also showed robust phosphorylation of EB2, whereas CDKL1, CDKL3, CDKL4, MAK and MOK did not (Fig. 5B). Next, we generated KD mutants of ICK and CDKL2, overexpressed these in HEK293T cells along with EB2 and assessed phosphorylation levels (Fig. 5C). EB2 phosphorylation was significantly increased in CDKL2 WT, ICK WT or CDKL5 WT transfected cells when compared to ICK KD, CDKL2 KD or CDKL5 KD, respectively (Fig. 5D–F). Thus, our data shows that ICK and CDKL2 can phosphorylate EB2 in HEK293T cells.

Next, we tested if these kinases could phosphorylate EB2 in neurons as well. For this purpose, FLAG-tagged ICK and CDKL2 WTs were overexpressed in *Cdkl5* KO mouse primary hippocampal neurons, which were then stained for EB2 pS222, and the fluorescence signal was compared between neurons expressing these kinases and untransfected neurons (Fig. 5G). *Cdkl5* WT mouse hippocampal neurons were also stained and the EB2 phosphorylation was quantified to compare endogenous EB2 signal. As anticipated, the EB2 pS222 levels in KO neurons are significantly lower when compared to WT neurons (Fig. 5H). Nevertheless, it is worth noting that the disparity in phosphorylation between *Cdkl5* WT and KO is less pronounced than our observations in Western blots (Fig. 1G). This phenomenon could be due to technical differences between immunocytochemistry and Western blotting. Non-specific signals in stainings cannot be readily distinguished from phospho-specific EB2 signal based on molecular weight, creating a higher background level. Whereas in Western blotting size separation enables us to distinguish signal at specific molecular weight. Interestingly, KO neurons expressing ICK and CDKL2 showed increased EB2 phosphorylation, comparable to KO neurons expressing CDKL5 (Fig. 5H), suggesting that ICK and CDKL2 could phosphorylate EB2 to the same extent as CDKL5 in neurons. EB2 pS222 was significantly increased in *Cdkl5* KO neurons overexpressing CDKL5 compared to untransfected *Cdkl5* KO neurons (Fig. 5H), suggesting an effect of overexpression. Furthermore, we found a significant decrease of total EB2 in neurons expressing CDKL5, but no changes were found in the ones expressing ICK or CDKL2 (Fig. 5I). Our results suggests that CDKL2 and ICK could be potential compensating kinases for CDKL5-dependent phospho-regulations.

### CDKL2 phosphorylates CDKL5 substrates in mouse brain

**Fig. 6:**
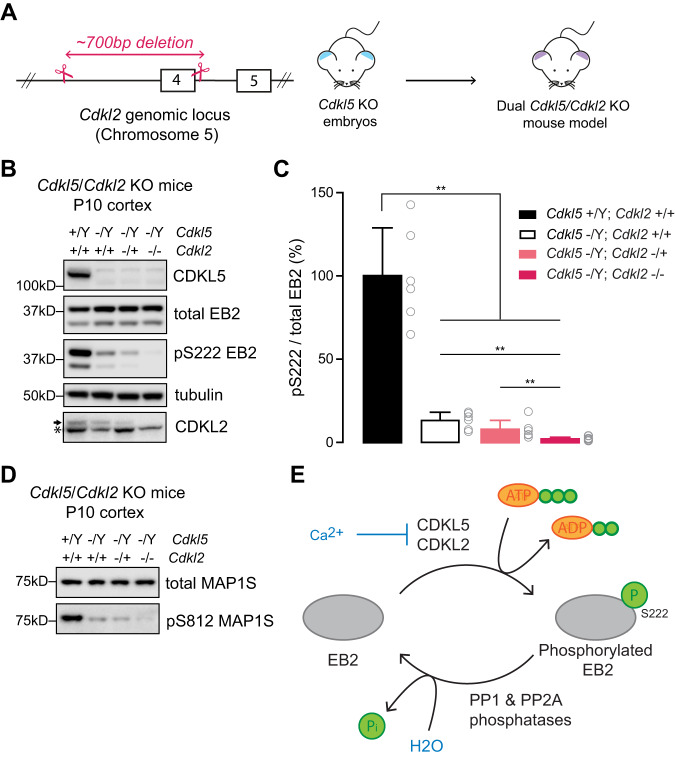
CDKL2 phosphorylates CDKL5 substrates in mouse brain. **A** Schematic of the generation of the dual *Cdkl5/Cdkl2* KO mouse model. An insert of 700 bp containing the exon 4 of *Cdkl2* was deleted from *Cdkl5* KO embryos using CRISPR-Cas9. **B** Western blot showing levels of CDKL5, total EB2 pS222, tubulin and CDKL2 in the cortex of P10 dual *Cdkl5/Cdkl2* KO animals. The arrow indicates CDKL2, [*] indicates a non-specific band. **C** Quantification of EB2 pS222 levels in the cortex of P10 *Cdkl5/Cdkl2* KO animals. *n* = 3 animals per genotype with 2 technical replicates. Mann–Whitney test. **p ≤ 0.01. **D** Western blot showing levels of total MAP1S and MAP1S pS812 in the cortex of P10 dual *Cdkl5/Cdkl2* KO animals. **E** Schematic encapsulating our findings. CDKL5 and CDKL2 phosphorylate EB2 on residue S222, a process modulated by calcium entry through NMDA receptors. Additionally, the dephosphorylation of EB2 S222 is attributed to the phosphatases PP1 and PP2A.

Overexpression of proteins in HEK293T cells and in primary neurons may not accurately reflect physiological conditions due to the high levels of kinase expression in these conditions. To address kinase compensation in a more physiological context, we decided to pursue dual kinase knockout mouse models. We first thoroughly examined the single-cell mRNA expression profiles of *Cdkl2* and *Ick* in both human and mouse brain using publicly available database [53, 54]. Our analysis revealed that while *Ick* mRNA was almost absent in neurons, a prominent *Cdkl2* mRNA expression was present in all neurons (Supplementary Fig S5). Based on this observation, we deduced that *Cdkl2* is more likely to be responsible for regulating the phosphorylation of EB2 in neurons. To test this, we generated a dual *Cdkl5/Cdkl2* KO mouse model by deleting *Cdkl2* exon 4 in *Cdkl5* KO embryos using Crispr/Cas9 (Fig. 6A). Dual *Cdkl5/Cdkl2* homozygous knockout mice were born at the expected mendelian ratios, indicating absence of sub-viability for dual deletion. Moreover, they displayed no overt physical abnormalities. We collected brains from dual *Cdkl5/Cdkl2* KO mice at P10 and compared the levels of EB2 pS222 with those of *Cdkl5* KO and C57BL/6 WT mice using Western blotting (Fig. 6B). We observed that EB2 pS222 level is significantly decreased in *Cdkl5* KO brains (Cdkl5 −/Y; Cdkl2 +/+) compared to WT (Cdkl5 +/Y; Cdkl2 +/+), as expected (Fig. 6B, C). The remaining EB2 phosphorylation in *Cdkl5* KO brains was approximately 15% (Fig. 6C), a level similar to that observed in *Cdkl5* KO cortical lysates in Fig. 1G. Interestingly, this remaining phosphorylation was further reduced in dual *Cdkl5/Cdkl2* KO brains (Cdkl5 −/Y; Cdkl2 −/−) (Fig. 6C). Additionally, we collected littermates that were heterozygote for *Cdkl2* (Cdkl5 −/Y; Cdkl2 −/+). While not statistically significant, the level of EB2 pS222 in these mice tended to be lower than in *Cdkl5* KO animals. Furthermore, the EB2 pS222 level in heterozygote mice (Cdkl5 −/Y; Cdkl2 −/+) was significantly higher than the dual *Cdkl5/Cdkl2* KO mice (Cdkl5 −/Y; Cdkl2 −/−) (Fig. 6C). These findings indicate that the level of EB2 phosphorylation is dependent on the dosage of CDKL2 in *Cdkl5* KO brains. We have noted that approximately 3-4% of EB2 phosphorylation remained in *Cdkl5/Cdkl2* dual knockout mice, indicating the involvement of additional kinase(s). Total EB2 levels were not changed in any of the genotypes and CDKL5 was lost in *Cdkl5* KO brains as expected (Supplementary Fig S6). Next, we tested if CDKL2 also phosphorylates MAP1S, another established CDKL5 substrate [11, 14] and found that, similar to EB2 pS222, MAP1S pS812 is reduced in dual *Cdkl5/2* KO brain (Fig. 6D).

Our results show that CDKL2 contributes approximately 15% of the total EB2 phosphorylation and CDKL5 phosphorylates approximately 80%, while a minor fraction of EB2 phosphorylation remains and can potentially be attributed to other kinases. pS812 MAP1S phosphorylation follows a similar pattern, indicating a compensatory role of CDKL2 for CDKL5 for multiple substrates at the molecular level. Altogether, our in vivo data reveals that CDKL2 is the previously unknown kinase, which is responsible for the majority of the remaining CDKL5-dependent phosphorylations in the brain.

## Discussion

### Cell-type specificity of CDKL5 expression and activity

We used a combination of methods to study CDKL5 localization including conditional knockout mice, primary neuronal cultures and publicly-available single-cell RNA sequencing data. We found that CDKL5 is expressed in glutamatergic and GABAergic neurons but not in astrocytes. It is possible, however, that the kinase activity is regulated in a distinct manner in different cell types. Therefore, we also checked the level of EB2 S222 phosphorylation using a phosphospecific antibody as a readout of CDKL5 activity and it shows a similar pattern as the CDKL5 expression. Indeed, CDKL5 is predominantly active in glutamatergic and GABAergic neurons, but not in astrocytes as measured by EB2 S222 phosphorylation. Our study suggests that CDKL5 is active in different types of neurons and it would be particularly interesting to determine if CDKL5 has different functions in different cell types. This could have a great impact on our understanding of the kinase, and why its mutations have such severe consequences. Several groups used *Cdkl5* conditional KO mice to investigate the roles of different neuronal populations in brain functions. In particular, it was shown that impaired learning primarily originates from the loss of CDKL5 in forebrain glutamatergic neurons, and autistic-like behaviors are primarily caused by the loss of CDKL5 in forebrain GABAergic neurons [22, 23]. However, the cell-type critical mechanisms for CDD pathology are unclear and additional studies are needed to elucidate them.

### Regulation of CDKL5 activity

Missense mutations in *CDKL5* gene that lead to CDD are predominantly located in the catalytic domain of the kinase [3, 8–10]. This suggests that regulation of its activity is crucial for brain development and finding its regulators could allow us to have a better understanding of the disease. In this study, we found that EB2 phosphorylation, and by extension CDKL5 activity, is regulated by entry of calcium through NMDA receptors (Fig. 6E). This finding corroborates with a previous study where CDKL5 activity was found to be downregulated upon KCl treatment [14], suggesting that CDKL5 activity is regulated by neuronal activity. We have also tested the contribution of synaptic and non-synaptic NMDARs to the reduction in CDKL5 activity and we found that the activation of synaptic NMDA receptors was responsible for regulating CDKL5. Studies in the past have shown that synaptic NMDA receptors are important for plasticity but also for cell survival [61, 63–65], suggesting that CDKL5 could be crucial for converting the effect of transient stimuli such as plasticity during neuronal activity.

### Regulation of EB2 dephosphorylation

Protein phosphorylation is a reversible and highly-dynamic mechanism where kinases, such as CDKL5, add a phosphate group to target proteins, and phosphatases remove it [66]. Much effort has been made to identify CDKL5 substrates, and several proteins including EB2 were found to be phosphorylated by CDKL5 [11, 14, 67]. However, little is known about the phosphatases that reverse the phosphorylation added by CDKL5. The mouse genome contains around 160 different phosphatases and the human genome has approximatively 200 [68, 69]. Considering that CDKL5 phosphorylates EB2 on a serine residue, we focused on the serine/threonine phosphatase family. Protein phosphatase 1 (PP1), protein phosphatase 2A (PP2A) and calcineurins (or also known as PP2B) are three families of phosphatases that have been studied heavily and are known to be expressed in the brain [70, 71]. In addition, specific inhibitors exist for these phosphatases [72–74]. OA is known to inhibit PP1 and PP2A phosphatases while CSA is a specific inhibitor of calcineurin phosphatases. We used these two compounds to treat primary neurons and assess the level of EB2 phosphorylation. The level of EB2 phosphorylation showed a significant and large increase upon treatment with OA, suggesting that EB2 is dephosphorylated by PP1 and PP2A phosphatases (Fig. 6E). On the contrary, treatment with CSA showed a small but significant decrease of EB2 phosphorylation. This finding suggests that calcineurins are not dephosphorylating EB2 but might actually downregulate its phosphorylation or deactivate CDKL5 itself.

### Kinases phosphorylating CDKL5 substrate EB2

We noticed that 18% of EB2 phosphorylation remained in *Cdkl5* full KO cerebral cortex, suggesting that CDKL5 is not the only kinase that can phosphorylate EB2. In order to verify that, we then used the phosphatase inhibitor OA which increases EB2 phosphorylation but, this time, in the absence of CDKL5 in primary neuronal cultures. We found that, even though CDKL5 was absent, EB2 phosphorylation was still significantly increased, emphasizing the existence of one or several kinases phosphorylating EB2. Similar to CDKL5, those kinases seem to be regulated by NMDA receptor activation as EB2 phosphorylation is reduced upon NMDA treatment in *Cdkl5* KO neurons. Finally, considering multiple lines of evidence suggesting that EB2 is phosphorylated by one or more kinases other than CDKL5, several potential candidates were investigated. We reasoned that kinases which share similarities with CDKL5 in the kinase domain would be more likely to have similar substrates and functions. CDKL5 is closely related to the RCK, GSK3 and CDKL families of kinases, and thus we considered them as candidates for the phosphorylation of EB2. GSK3s are well studied kinases and CDKL5 consensus phosphorylation site, RPXS*, is distinct from the GSK3 consensus sequence [62]. Therefore, we decided to focus on RCK and CDKL kinases, in order to determine if they could phosphorylate EB2 as well. We were able to show that ICK and CDKL2 do phosphorylate EB2, using overexpression in HEK293T cells and mouse primary neurons (Fig. 6E).

### CDKL2 is a potential CDD therapeutic target

Through meticulous examination of EB2 S222 phosphorylation under varying neuronal activity conditions and treatments with phosphatase inhibitors, our study has determined, for the first time, the presence of a potential compensatory kinase within neurons that could assume the role of CDKL5 activity. Following this observation, we were able to pinpoint this kinase to be CDKL2, via a cellular screen and creation of a novel dual *Cdkl5/Cdkl2* KO mouse model. Our results revealed a significant decrease of EB2 and MAP1S phosphorylations in the dual KO compared to the *Cdkl5* KO mice, providing robust evidence that CDKL2 plays a role in phosphorylating CDKL5 substrates in vivo.

CDKL2 exhibits a remarkably similar expression pattern to CDKL5. Specifically, CDKL2 is expressed from postnatal day 3 (P3) in mice and gradually increases, reaching a peak at P28 [75]. This pattern resembles CDKL5’s expression, which plateaues between P14 and P28 [44, 76, 77]. CDKL2 is detected in various brain regions, including olfactory bulb, cerebral cortex and hippocampus [75], similar to CDKL5 [18, 43, 44]. Despite the presence of CDKL2 in the brain of CDKL5 knockout mice, substrate phosphorylations (EB2 and MAP1S) remain significantly reduced and CDKL5 KO mice exhibit learning and memory deficits as well as stereotypical behaviors [16], indicating that the existing CDKL2 levels are not fully capable of compensating for the loss of CDKL5 in mice. It is interesting to note that *Cdkl2* KO mice exhibit mild impairments in learning tasks [78], suggesting a potential collaborative role of CDKL5 and CDKL2 in brain development, crucial for learning and behavior. CDKL5 is known to be present in synapses [37], CDKL2’s subcellular localization is yet to be studied. For a comprehensive understanding of the functional interplay between these two kinases, a rigorous behavioral and physiological characterization of the dual *Cdkl5/Cdkl2* mouse model would be necessary.

This study constitutes a significant advancement in the identification of CDKL2 as a potential therapeutic target for CDKL5 deficiency disorder. The presence of *CDKL2* mRNA in neurons (Supplementary Fig. S5) presents a novel possibility of targeting *CDKL2* mRNA or protein in neurons to increase its expression and/or activity. As of today, a cure for CDD patients remains elusive, with only symptomatic treatments being employed. Our study opens new possibilities for much-needed and urgent therapeutic strategies involving upregulating of CDKL2 function in CDD patients.

## Supplementary information

Supplemental Material

## Data Availability

Western blot images and fluorescent images that support the findings of this study are openly available in Figshare 10.25418/crick.24962133.
