# Supplementary material for: Cell type-specific expression, regulation and compensation of CDKL5 activity in mouse brain
Source: Mol Psychiatry. 2024 Feb 8;29(6):1844–56. doi: 10.1038/s41380-024-02434-7 (PMC11371643; doi:10.1038/s41380-024-02434-7)
Supplement: Supplementary file 1 — Supplemental Material [file 41380_2024_2434_MOESM1_ESM.pdf]

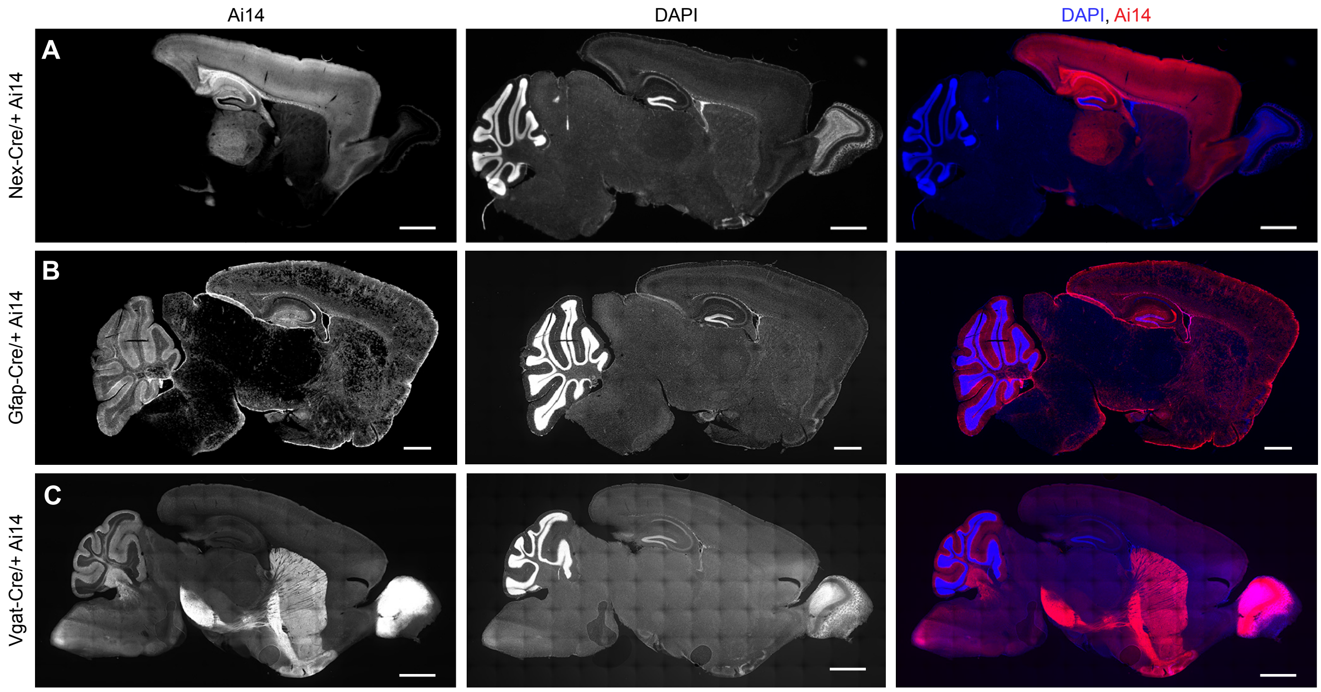

Supplementary Figure 1. Cre mediated recombination in P20 Nex-Cre, Gfap-Cre and Vgat-Cre mouse brain by Ai14 reporter. (A-C) Sagittal vibratome section of P20 Nex-Cre, Gfap-Cre Vgat-Cre Ai14 mouse brain. Expression of reporter is detected in cerebral cortex and hippocampus for Nex-cre Ai14 brain. However, Ai14 is expressed in the whole brain for Gfap-Cre Ai14 brain. Finally, Ai14 is mainly detected in the striatum and hypothalamus for Vgat-Cre Ai14 brains. Scale bars is 1 mm.

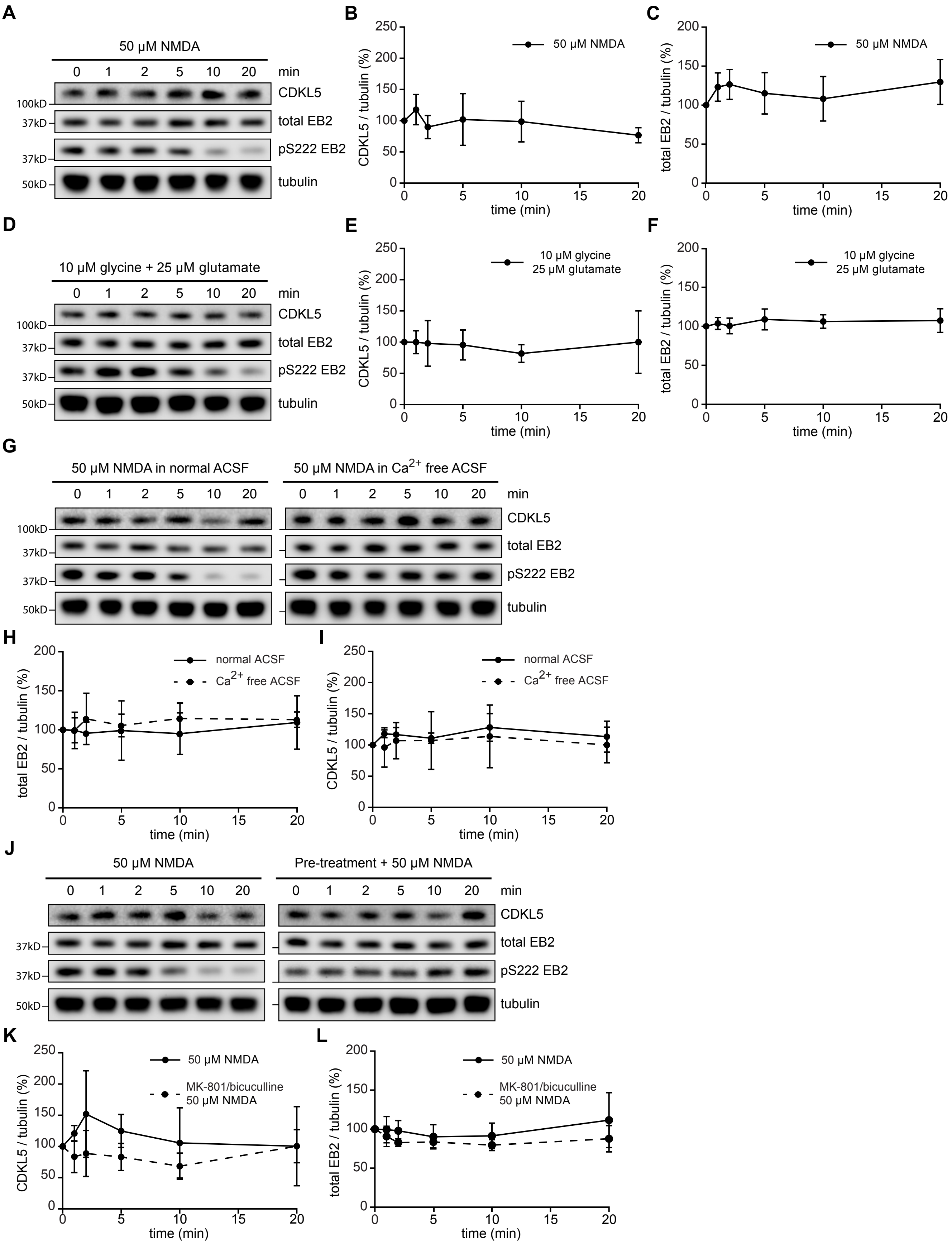
A
50 μM NMDA
0 1 2 5 10 20 min
CDKL5
total EB2
pS222 EB2
tubulin
100kD
37kD
37kD
50kD
B
50 μM NMDA
CDKL5 / tubulin (%)
time (min)
C
50 μM NMDA
total EB2 / tubulin (%)
time (min)
D
10 μM glycine + 25 μM glutamate
0 1 2 5 10 20 min
CDKL5
total EB2
pS222 EB2
tubulin
E
10 μM glycine
25 μM glutamate
CDKL5 / tubulin (%)
time (min)
F
10 μM glycine
25 μM glutamate
total EB2 / tubulin (%)
time (min)
G
50 μM NMDA in normal ACSF
50 μM NMDA in Ca2+ free ACSF
0 1 2 5 10 20
0 1 2 5 10 20 min
CDKL5
total EB2
pS222 EB2
tubulin
H
normal ACSF
Ca2+ free ACSF
total EB2 / tubulin (%)
time (min)
I
normal ACSF
Ca2+ free ACSF
CDKL5 / tubulin (%)
time (min)
J
50 μM NMDA
Pre-treatment + 50 μM NMDA
0 1 2 5 10 20
0 1 2 5 10 20 min
CDKL5
total EB2
pS222 EB2
tubulin
K
50 μM NMDA
MK-801/bicuculline
50 μM NMDA
CDKL5 / tubulin (%)
time (min)
L
50 μM NMDA
MK-801/bicuculline
50 μM NMDA
total EB2 / tubulin (%)
time (min)

Supplementary Figure 2. CDKL5 and EB2 expression upon different NMDA treatments. (A) Western blots showing expression of CDKL5, EB2 and tubulin, and level of EB2 pS222 upon 50 µM NMDA treatment of DIV14 rat primary cortical neurons. **(B-C)** Quantification of CDKL5 and total EB2 expression respectively upon 50 µM NMDA treatment of DIV14 rat primary cortical neurons. Welch's t-test. n = 3 replicates. **(D)** Western blots showing expression of CDKL5, EB2 and tubulin, and level of EB2 pS222 upon 10 µM glycine and 25 µM glutamate treatment of DIV15 rat primary cortical neurons. **(E-F)** Quantification of CDKL5 and total EB2 expression respectively upon 10 µM glycine and 25 µM glutamate treatment of DIV15 rat primary cortical neurons. Welch's t-test. n = 3 replicates. **(G)** Western blots showing expression of CDKL5, EB2 and tubulin, and level of EB2 pS222 upon 50 µM NMDA treatment of DIV15 rat primary cortical neurons in normal ACSF and in calcium-free ACSF. **(H-I)** Quantification of CDKL5 and total EB2 expression respectively upon 50 µM NMDA treatment of DIV15 rat primary cortical neurons in normal ACSF and in calcium free ACSF. Dunnett's multiple comparisons test. n = 4 replicates. **(J)** Western blots showing expression of CDKL5, EB2 and tubulin, and level of EB2 pS222 upon 10 µM MK801 and 50 µM Bicuculline pre-treatment (10 minutes) and 50 µM NMDA treatment of DIV15 rat primary cortical neurons. **(K-L)** Quantification of CDKL5 and total EB2 expression respectively upon 10 µM MK801 and 50 µM Bicuculline pre-treatment (10 minutes) and 50 µM NMDA treatment of DIV15 rat primary cortical neurons. Dunnett's multiple comparisons test. n = 4 replicates.

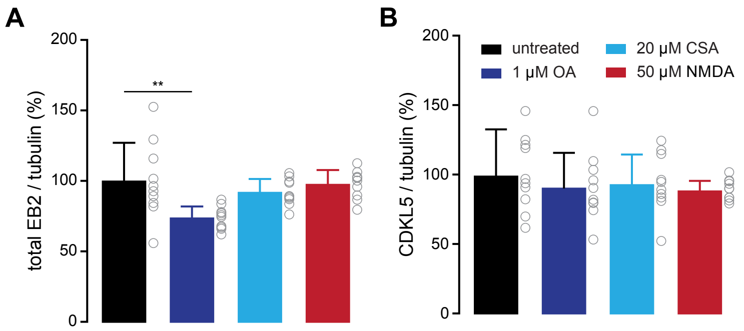

Supplementary Figure 3. EB2 and CDKL5 expression upon OA, CSA and NMDA treatment in rat primary neurons. (A-B) Quantification of respectively total EB2 and CDKL5 expression upon 1 μM okadaic acid (OA) for 40 minutes, 20 μM cyclosporin A (CSA) for 40 minutes and 50 μM NMDA treatment for 20 minutes in DIV14-15 rat primary cortical culture. Mann-Whitney test. n = 5 with 2 technical replicates per treatment. **$p \leq 0.01$.

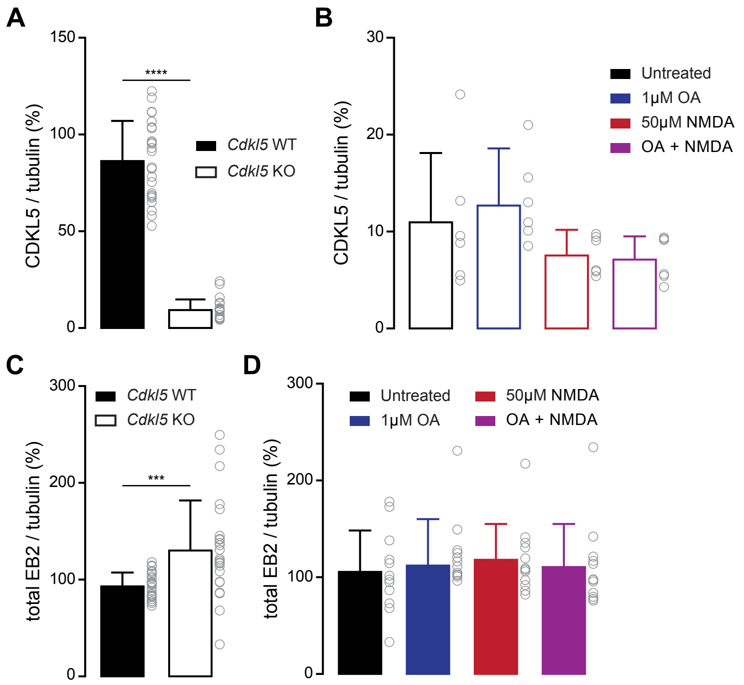

Supplementary Figure 4. EB2 and CDKL5 expression upon NMDA and OA treatments in *Cdkl5* mouse primary culture. (A, C) Quantification of respectively CDKL5 and total EB2 expression in DIV8 mouse *Cdkl5* WT and KO primary cortical culture upon 1 μM OA treatment for 40 minutes, 50 μM NMDA treatment for 20 minutes and a combination of OA and NMDA treatments (1 μM OA treatment for 20 minutes followed by 20 minutes of OA + NMDA). The data points of the different treatments were pulled together for each genotype. Mann-Whitney test. n = 12 with 2 technical replicates per treatment. ***p≤ 0.005. ****p≤0.001. **(B)** Quantification of CDKL5 expression in DIV8 mouse *Cdkl5* KO primary cortical culture upon 1 μM OA treatment for 40 minutes, 50 μM NMDA treatment for 20 minutes and a combination of OA and NMDA treatments. Mann-Whitney test. n = 3 with 2 technical replicates per treatment. **(D)** Quantification of total EB2 expression in DIV8 mouse *Cdkl5* WT and KO primary cortical culture upon 1 μM OA treatment for 40 minutes, 50 μM NMDA treatment for 20 minutes and a combination of OA and NMDA treatments. The data points of the two genotypes were pulled together for each genotype. Mann-Whitney test. n = 6 with 2 technical replicates per treatment.

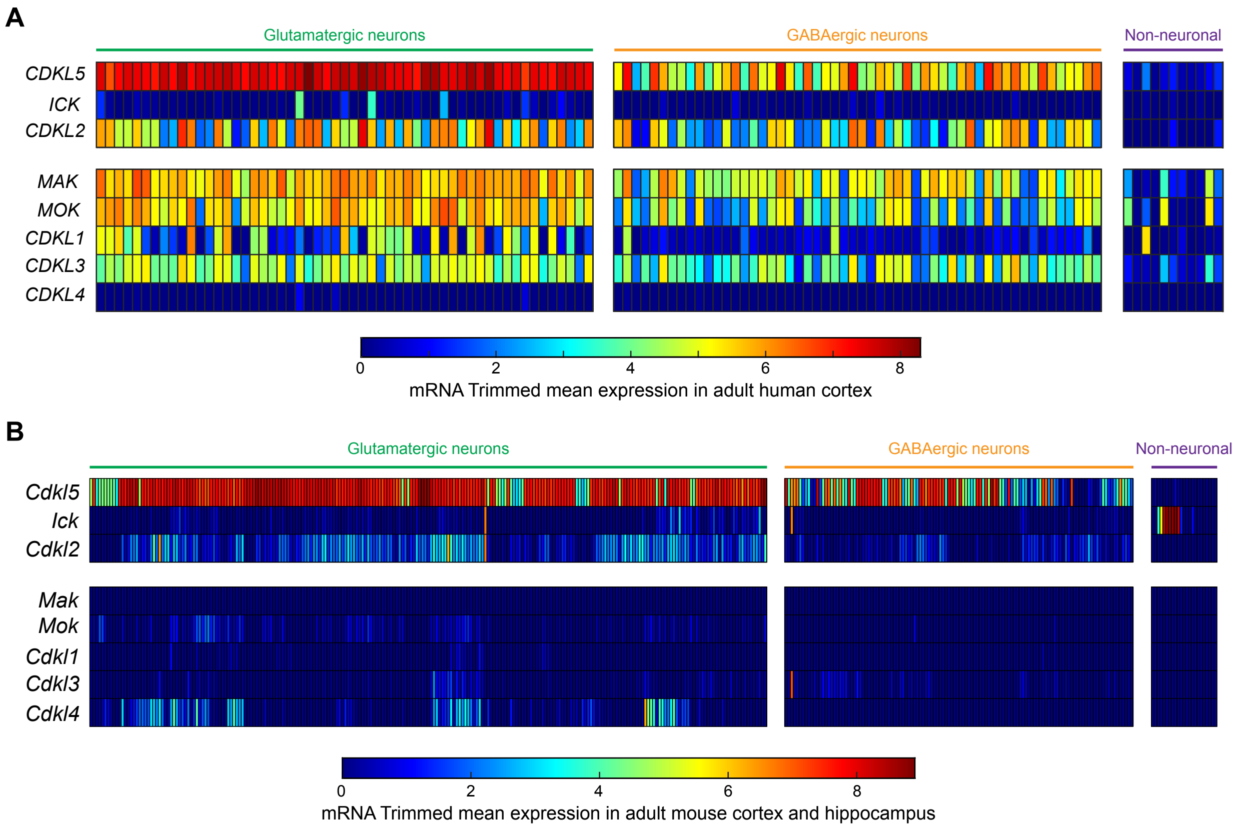

Supplementary Figure 5. mRNA expression of *CDKL1-5, ICK, MAK* and *MOK* in mouse and human adult brain. (A) mRNA expression of *CDKL1, CDKL2, CDKL3, CDKL4, CDKL5, ICK, MAK* and *MOK* in multiple cortical areas of human brain from single-cell RNA sequencing data (Hodge, Bakken et al. 2019, Hodge, Bakken et al. 2019). **(B)** mRNA expression of *Cdkl1, Cdkl2, Cdkl3, Cdkl4, Cdkl5, Ick, Mak* and *Mok* in mouse adult isocortex and hippocampus from single-cell RNA sequencing data (Yao, van Velthoven et al. 2020, Yao, van Velthoven et al. 2021).

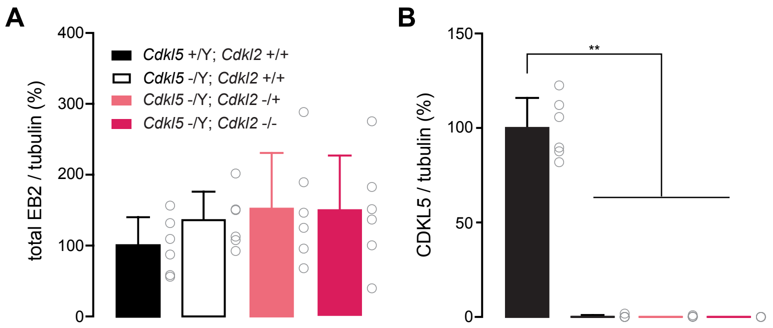

Supplementary Figure 6. CDKL2 phosphorylates EB2 in the mouse brain. (A) Quantification of total EB2 levels in cortex of P10 *Cdkl5/Cdkl2* KO mice. n = 3 per genotype with 2 technical replicates. Mann-Whitney test. **p≤0.01. **(B)** Quantification of CDKL5 levels in cortex of P10 *Cdkl5/Cdkl2* KO mice. n = 3 per genotype with 2 technical replicates. Mann-Whitney test. **p≤0.01.
